# Association of the prealbumin–albumin nutritional index with short-term mortality in hospitalized patients with traumatic brain injury

**DOI:** 10.3389/fnut.2026.1910413

**Published:** 2026-09-03

**Authors:** Hong Huang, Shaorong Yi, Jinrong Wang, Hua Liu, Chengyuan Ji

**Affiliations:** 1 Department of Neurosurgery, Affiliated Kunshan Hospital of Jiangsu University, Kunshan, Jiangsu, China; 2 Department of Neurosurgery, The First Affiliated Hospital of Soochow University, Suzhou, Jiangsu, China

**Keywords:** albumin, prealbumin, prealbumin–albumin nutritional index, short-term mortality, traumatic brain injury

## Abstract

**Methods:**

This single-center retrospective cohort study included 2,221 patients with TBI admitted between March 29, 2018, and September 23, 2024. PANI was calculated as serum prealbumin (mg/L) multiplied by albumin (g/L) and was divided by 1,000 for analysis. The first available laboratory measurements after admission were used. The primary and secondary outcomes were all-cause in-hospital mortality and 7-day in-hospital mortality, respectively. Associations were assessed using Cox proportional hazards models. Discrimination was evaluated using receiver operating characteristic curves and pairwise comparisons of areas under the curve (AUCs).

**Results:**

Among 2,221 patients, 78 (3.5%) died during hospitalization and 47 (2.1%) died in hospital within 7 days of admission. In the primary adjusted model (Model 3), each 1-unit increase in PANI/1,000 was associated with lower hazards of in-hospital mortality (HR, 0.613; 95% CI, 0.560–0.671) and 7-day in-hospital mortality (HR, 0.640; 95% CI, 0.574–0.714). Corresponding HRs in the supplementary extended baseline model (Model 4) were 0.653 (95% CI, 0.593–0.720) and 0.690 (95% CI, 0.616–0.773), respectively. In the complete-case sensitivity analysis additionally incorporating body mass index and systolic blood pressure, the corresponding HRs were 0.562 (95% CI, 0.466–0.678) and 0.677 (95% CI, 0.528–0.870). PANI alone yielded AUCs of 0.856 (95% CI, 0.810–0.902) and 0.837 (95% CI, 0.774–0.901), respectively.

**Conclusion:**

Lower PANI was associated with higher in-hospital and 7-day in-hospital mortality in patients with TBI. However, the single-center design, potential selection bias, residual confounding, and absence of key neurological and imaging severity variables preclude conclusions regarding its clinical utility. The potential clinical value of PANI can only be evaluated after validation in multicenter prospective studies incorporating complete neurological and imaging assessments.

## 1 Background

Traumatic brain injury (TBI) is a major cause of death and disability worldwide. Mortality risk varies substantially according to injury severity, age, physiologic derangement, and the presence of extracranial injury. Early identification of patients at increased risk may help guide monitoring and communication; however, any new biomarker should be evaluated as an adjunct to, rather than a replacement for, established clinical assessment and validated TBI prognostic models (1–5).

Composite indices derived from routine hematologic and biochemical tests have been investigated as prognostic markers in several clinical settings. The prognostic nutritional index (PNI), hemoglobin-albumin-lymphocyte-platelet (HALP) score, and albumin-to-prealbumin ratio (APR) combine markers of protein status, inflammation, or hematologic disturbance (6–11). Albumin and prealbumin have also been associated with outcomes after TBI (12–14). These proteins are negative acute-phase reactants, however, and their concentrations may reflect inflammation, fluid balance, capillary permeability, hepatic synthesis, and disease severity in addition to nutritional status. Evidence comparing albumin- and prealbumin-based indices for short-term mortality in TBI remains limited.

We therefore examined the associations of five composite indices with in-hospital mortality and 7-day in-hospital mortality in a retrospective cohort of hospitalized patients with TBI. We evaluated a new index, PANI, defined as the product of admission prealbumin and albumin concentrations, and compared its discrimination with PNI, HALP, APR, and the composite prealbumin nutritional index (CPNI). We hypothesized that lower PANI would be associated with higher short-term mortality. Because PANI was developed and evaluated in the same dataset, all performance comparisons were considered exploratory.

## 2 Materials and methods

### 2.1 Study framework and subjects

**Figure 1:**
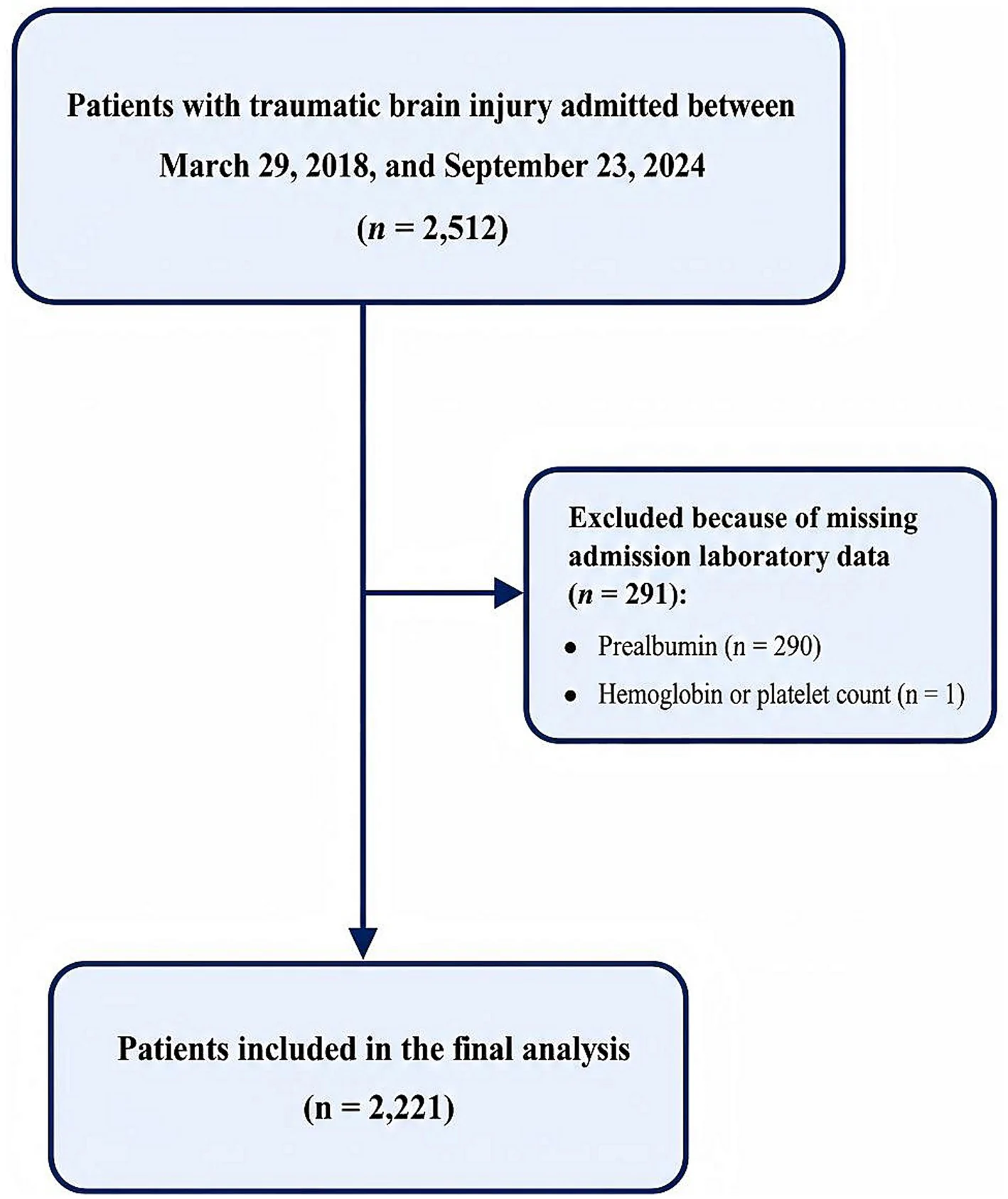
Flowchart of patient selection and study inclusion. The screening process initially identified 2,512 patients admitted with traumatic brain injury (TBI) between March 29, 2018, and September 23, 2024. After excluding 291 patients with missing admission laboratory data, including missing prealbumin values (*n* = 290) and missing hemoglobin or platelet count (*n* = 1), a final cohort of 2,221 patients was included in the analysis. TBI, traumatic brain injury.

This single-center retrospective cohort study was conducted in the Department of Neurosurgery at Affiliated Kunshan Hospital of Jiangsu University. TBI was diagnosed by a neurosurgeon based on traumatic abnormalities identified on cranial computed tomography and the clinical history of head trauma. We screened 2,512 consecutive patients admitted with TBI between March 29, 2018, and September 23, 2024. Patients were excluded if the first available post-admission measurement of albumin, prealbumin, hemoglobin, lymphocyte count, or platelet count was missing. After 291 exclusions, 2,221 patients were included (Figure 1). Of the 291 excluded patients, 290 lacked a prealbumin measurement and one had missing hemoglobin or platelet count. Because prealbumin accounted for nearly all laboratory-related exclusions, we additionally compared patients with available versus missing prealbumin measurements within the 2,512-patient source cohort to assess possible selective measurement and selection bias (Supplementary Table S8).

### 2.2 Exposures and outcomes

The five indices were calculated from the first available post-admission laboratory measurements: PNI = albumin (g/L) + 5 × lymphocyte count (10^9^/L); HALP = hemoglobin (g/L) × albumin (g/L) × lymphocyte count (10^9^/L)/platelet count (10^9^/L); APR = albumin (g/L)/prealbumin (mg/L); PANI = prealbumin (mg/L) × albumin (g/L); and CPNI = prealbumin (mg/L) × albumin (g/L) × lymphocyte count (10^9^/L). PANI and CPNI were divided by 1,000 for analysis. PANI/1,000 was also categorized into tertiles for descriptive and exploratory analyses. The compositional architecture of the five indices is illustrated in Supplementary Figure S1. The exact interval from admission to blood sampling and the temporal relationships between sampling and albumin infusion, blood transfusion, or major surgery could not be retrospectively ascertained.

The primary outcome was all-cause in-hospital mortality. The secondary outcome was 7-day in-hospital mortality, defined as death occurring during hospitalization within 7 days after admission. Time to event was calculated from admission. For the in-hospital mortality analysis, patients discharged alive were censored on the date of discharge. For the 7-day time-to-event analysis, patients discharged alive before day 7 were censored on the date of discharge, whereas patients remaining alive in hospital were censored at day 7. For descriptive and receiver operating characteristic analyses, patients without an in-hospital death within 7 days, including those discharged alive before day 7, were classified as non-events. Outcome ascertainment used discharge summaries and inpatient disposition records. Post-discharge mortality was unavailable.

### 2.3 Covariates

Covariates were selected *a priori* on the basis of clinical relevance and previous literature (15, 16): age, sex, diabetes, hypertension, tracheotomy, mechanical ventilation, craniotomy or brain surgery, and inferior vena cava filter placement. Because several treatment variables occur after admission and may lie on the causal pathway between injury severity and death, models including these variables should be interpreted as adjusted association models rather than causal models. Important measures of TBI severity, including the Glasgow Coma Scale score, pupillary reactivity, computed tomography findings, hypotension, hypoxia, and extracranial injury severity, were unavailable and therefore not adjusted for.

To further address residual confounding using additional variables available in the database, a supplementary extended baseline model (Model 4) included age, sex, diabetes, hypertension, other injury, alanine aminotransferase, creatinine, and international normalized ratio. “Other injury” was recorded as a binary variable indicating the presence of a documented concomitant injury in addition to the index TBI; detailed anatomical location and injury severity were not available in the source database. Body mass index and systolic blood pressure were additionally evaluated in a complete-case sensitivity analysis because of substantial missingness. Missing-data patterns in the manuscript source cohort and final analytical cohort are summarized in Supplementary Table S6.

### 2.4 Statistical analyses

Continuous variables were summarized as mean ± standard deviation and compared using Welch’s two-sample *t* test. Categorical variables were reported as number (percentage) and compared using Pearson’s chi-square test or Fisher’s exact test, as appropriate. Standardized mean differences with 95% confidence intervals were also calculated. For the prealbumin missingness analysis, patients with available versus missing prealbumin measurements in the source cohort were compared descriptively using Welch’s two-sample *t* tests for continuous variables and Pearson’s chi-square or Fisher’s exact tests for categorical variables, as appropriate; standardized mean differences were also reported. This analysis was intended to assess possible selective prealbumin measurement and was not used to infer the mechanism or direction of missing-data bias.

Discrimination for each outcome was assessed using ROC curves, AUCs with 95% CIs, and pairwise AUC comparisons. Cutoffs were selected by the maximum Youden index and were reported on the analysis scale shown for each index; PANI and CPNI values were divided by 1,000. Because cutoffs were selected in the development cohort, sensitivity and specificity estimates are optimistic and require external validation. Because the cutoff values were derived from the same development cohort, they were considered exploratory development-cohort estimates requiring external validation. Pairwise AUC comparisons were performed using two-sided paired DeLong tests. Because several nutritional indices shared common laboratory components, pairwise Spearman correlation coefficients and variance inflation factors were calculated. The five indices were evaluated in separate models and were not simultaneously entered into the primary Cox regression models.

Cox proportional hazards regression was used to estimate HRs and 95% CIs for PANI/1,000. Model 1 was unadjusted; Model 2 was adjusted for age and sex; and the primary adjusted model (Model 3) additionally included diabetes, hypertension, tracheotomy, mechanical ventilation, craniotomy or brain surgery, and inferior vena cava filter placement. The supplementary extended baseline model (Model 4) was adjusted for age, sex, diabetes, hypertension, other injury, alanine aminotransferase, creatinine, and international normalized ratio. The proportional hazards assumption was assessed using scaled Schoenfeld residuals for individual covariates and global Schoenfeld tests. Tertile-specific and restricted cubic spline analyses were exploratory. All restricted cubic spline Cox models were adjusted using the Model 3 covariate set (age, sex, diabetes, hypertension, tracheotomy, mechanical ventilation, craniotomy or brain surgery, and inferior vena cava filter placement). The supplementary extended baseline model (Model 4) was used only for the conventional Cox analyses and was not used for the restricted cubic spline analyses. To assess the robustness of the spline findings, three-knot models with knots at the 10th, 50th, and 90th percentiles of PANI/1,000 were additionally fitted. For transparency, the original exploratory four-knot PANI models, with knots at the 5th, 35th, 65th, and 95th percentiles, were retained only in Supplementary Table S7. Because the upper-tail curvature was potentially influenced by a single death event with an unusually high PANI value, an influence sensitivity analysis was performed after exclusion of this observation while retaining the same three-knot locations. Overall association and nonlinearity were evaluated using likelihood-ratio tests.

Effect modification was formally assessed using multiplicative interaction terms between continuous PANI/1,000 and each subgroup variable. When the subgroup variable was itself included in the Model 3 covariate set, that variable was omitted from the corresponding subgroup-specific and interaction models. Because multiple interaction tests were performed, Benjamini–Hochberg false discovery rate (BH-FDR) correction was applied separately within each outcome across evaluable interaction tests. The interaction involving inferior vena cava filter placement was considered not evaluable because of sparse events and was excluded from the multiplicity correction. Subgroup analyses were considered exploratory.

Analyses were performed using R version 4.5.2 (R Foundation for Statistical Computing, Vienna, Austria). All tests were two-sided. *p* < 0.05 was considered statistically significant. Pairwise AUC comparisons and other secondary analyses were considered exploratory. Interaction tests were additionally adjusted for multiple testing using the BH-FDR procedure as described above. The study was reported in accordance with the STROBE statement.

## 3 Results

### 3.1 Patient characteristics

A total of 2,221 patients with TBI were included in the final analysis, of whom 78 died during hospitalization and 47 died in hospital within 7 days of admission. The baseline characteristics of the study participants are summarized in Table 1.

**Table 1:** Baseline characteristics according to in-hospital and 7-day in-hospital mortality.

Characteristic	Missing, *n*	In-hospital mortality				7-day in-hospital mortality			
		Survivors (*n* = 2,143)	Nonsurvivors (*n* = 78)	SMD (95% CI)	*p* value	No 7-day in-hospital death (*n* = 2,174)	7-day in-hospital death (*n* = 47)	SMD (95% CI)	*p* value
Total	0	2,143	78			2,174	47		
Age, years	0	50.43 ± 17.36	53.47 ± 16.58	0.18 (−0.05, 0.41)	0.116	50.40 ± 17.33	57.17 ± 16.55	0.40 (0.11, 0.69)	0.008
Body mass index, kg/m^2^	626	23.53 ± 3.63	22.40 ± 2.86	0.34 (−0.04, 0.72)	0.054	23.51 ± 3.63	22.51 ± 3.10	0.30 (−0.27, 0.87)	0.286
Systolic blood pressure, mmHg	614	136.40 ± 22.00	135.19 ± 37.51	0.04 (−0.19, 0.27)	0.781	136.36 ± 22.22	135.57 ± 41.24	0.02 (−0.27, 0.32)	0.897
Albumin, g/L	0	38.94 ± 5.79	25.99 ± 11.37	1.44 (1.21, 1.66)	<0.001	38.74 ± 6.13	26.65 ± 11.40	1.32 (1.03, 1.61)	<0.001
Prealbumin, mg/L	0	234.31 ± 67.25	169.64 ± 51.28	1.08 (0.85, 1.31)	<0.001	233.25 ± 67.59	176.40 ± 53.23	0.93 (0.64, 1.22)	<0.001
Hemoglobin, g/L	0	126.86 ± 24.15	97.96 ± 33.17	1.00 (0.77, 1.22)	<0.001	126.43 ± 24.51	98.40 ± 34.54	0.94 (0.65, 1.23)	<0.001
Lymphocyte count, ×10^9^/L	0	1.20 ± 0.63	1.46 ± 1.73	0.20 (−0.03, 0.42)	0.193	1.21 ± 0.69	1.29 ± 0.88	0.10 (−0.19, 0.39)	0.539
Platelet count, ×10^9^/L	0	187.57 ± 65.97	139.16 ± 170.57	0.37 (0.15, 0.60)	0.015	187.31 ± 72.21	119.35 ± 64.92	0.99 (0.70, 1.28)	<0.001
Alanine aminotransferase, U/L	0	29.31 ± 37.56	34.19 ± 33.29	0.14 (−0.09, 0.36)	0.209	29.46 ± 37.70	30.36 ± 20.38	0.03 (−0.26, 0.32)	0.772
Aspartate aminotransferase, U/L	0	36.55 ± 43.13	58.65 ± 51.96	0.46 (0.24, 0.69)	<0.001	37.03 ± 43.78	51.15 ± 34.82	0.36 (0.07, 0.65)	0.009
Creatinine, μmol/L	0	63.77 ± 40.47	73.83 ± 30.55	0.28 (0.05, 0.51)	0.006	63.92 ± 40.40	73.32 ± 28.59	0.27 (−0.02, 0.56)	0.032
Urea nitrogen, mmol/L	0	5.24 ± 2.09	5.60 ± 1.86	0.19 (−0.04, 0.41)	0.092	5.24 ± 2.09	5.86 ± 1.73	0.33 (0.04, 0.61)	0.019
International normalized ratio	70	1.02 ± 0.16	1.31 ± 0.29	1.24 (1.01, 1.47)	<0.001	1.02 ± 0.17	1.32 ± 0.31	1.19 (0.90, 1.49)	<0.001
PNI	0	44.93 ± 6.96	33.27 ± 15.54	0.97 (0.74, 1.20)	<0.001	44.77 ± 7.40	33.08 ± 12.50	1.14 (0.85, 1.43)	<0.001
CPNI/1,000	0	11.42 ± 7.59	7.28 ± 13.52	0.38 (0.15, 0.60)	0.009	11.38 ± 7.90	6.34 ± 6.35	0.70 (0.41, 0.99)	<0.001
HALP	0	34.51 ± 22.27	35.04 ± 60.83	0.01 (−0.21, 0.24)	0.939	34.44 ± 22.25	38.43 ± 76.70	0.07 (−0.22, 0.36)	0.723
APR	0	0.18 ± 0.07	0.16 ± 0.07	0.28 (0.05, 0.50)	0.024	0.18 ± 0.07	0.16 ± 0.07	0.32 (0.03, 0.61)	0.038
PANI/1,000	0	9.29 ± 3.40	4.66 ± 3.11	1.42 (1.19, 1.65)	<0.001	9.22 ± 3.45	4.94 ± 3.30	1.27 (0.98, 1.56)	<0.001
Sex	0			0.02 (−0.20, 0.25)	0.830			0.02 (−0.27, 0.31)	0.908
Male		1,535 (71.63%)	55 (70.51%)			1,556 (71.57%)	34 (72.34%)		
Female		608 (28.37%)	23 (29.49%)			618 (28.43%)	13 (27.66%)		
Other injury	0			0.28 (0.06, 0.51)	0.005			0.10 (−0.19, 0.39)	0.492
No		1,900 (88.66%)	61 (78.21%)			1,921 (88.36%)	40 (85.11%)		
Yes		243 (11.34%)	17 (21.79%)			253 (11.64%)	7 (14.89%)		
Diabetes	0			0.07 (−0.16, 0.29)	0.589			0.15 (−0.14, 0.43)	0.291
No		2,038 (95.10%)	73 (93.59%)			2,068 (95.12%)	43 (91.49%)		
Yes		105 (4.90%)	5 (6.41%)			106 (4.88%)	4 (8.51%)		
Hypertension	0			0.19 (−0.03, 0.42)	0.139			0.03 (−0.25, 0.32)	0.818
No		1,888 (88.10%)	73 (93.59%)			1,919 (88.27%)	42 (89.36%)		
Yes		255 (11.90%)	5 (6.41%)			255 (11.73%)	5 (10.64%)		
Tracheotomy	0			0.13 (−0.10, 0.36)	0.295			0.07 (−0.22, 0.36)	0.643
No		1,864 (86.98%)	71 (91.03%)			1,893 (87.07%)	42 (89.36%)		
Yes		279 (13.02%)	7 (8.97%)			281 (12.93%)	5 (10.64%)		
Mechanical ventilation	0			0.11 (−0.12, 0.33)	0.306			0.12 (−0.17, 0.41)	0.386
No		1,989 (92.81%)	70 (89.74%)			2,017 (92.78%)	42 (89.36%)		
Yes		154 (7.19%)	8 (10.26%)			157 (7.22%)	5 (10.64%)		
Craniotomy/brain surgery	0			0.05 (−0.18, 0.27)	0.691			0.05 (−0.24, 0.34)	0.724
No		1,555 (72.56%)	55 (70.51%)			1,577 (72.54%)	33 (70.21%)		
Yes		588 (27.44%)	23 (29.49%)			597 (27.46%)	14 (29.79%)		
Orthopedic surgery	0			0.12 (−0.10, 0.35)	0.730			0.25 (−0.04, 0.54)	0.400
No		2,077 (96.92%)	77 (98.72%)			2,107 (96.92%)	47 (100.00%)		
Yes		66 (3.08%)	1 (1.28%)			67 (3.08%)	0 (0.00%)		
IVC filter	0			0.12 (−0.10, 0.35)	0.181			0.14 (−0.15, 0.43)	0.222
No		2,104 (98.18%)	75 (96.15%)			2,134 (98.16%)	45 (95.74%)		
Yes		39 (1.82%)	3 (3.85%)			40 (1.84%)	2 (4.26%)		

Values are mean ± standard deviation or *n* (%), unless otherwise indicated.

In the 2,512-patient source cohort, prealbumin measurements were available for 2,222 patients and missing for 290 patients (11.5%). Patients without available prealbumin measurements had higher in-hospital mortality than those with available measurements (40/290 [13.8%] vs. 78/2,222 [3.5%]; *p* < 0.001) and higher 7-day in-hospital mortality (32/290 [11.0%] vs. 47/2,222 [2.1%]; *p* < 0.001) (Supplementary Table S8).

Patients who died during hospitalization had lower admission albumin, prealbumin, hemoglobin, platelet count, PNI, CPNI/1,000, and PANI/1,000 values than survivors. The between-group *p* values were <0.001 for albumin, prealbumin, hemoglobin, PNI, and PANI/1,000; 0.015 for platelet count; and 0.009 for CPNI/1,000. PANI/1,000 showed the largest standardized difference between groups (SMD, 1.42). APR also differed between groups (*p* = 0.024), whereas HALP did not (*p* = 0.939). These unadjusted comparisons describe group differences and should not be interpreted as evidence of independent prognostic effects.

Patients who died in hospital within 7 days were older than those without a 7-day in-hospital death (*p* = 0.008) and had lower albumin, prealbumin, hemoglobin, platelet count, PNI, CPNI/1,000, and PANI/1,000 values (all *p* < 0.001). PANI/1,000 again showed the largest standardized difference between groups (SMD, 1.27). APR also differed between groups (*p* = 0.038), whereas HALP did not (*p* = 0.723).

### 3.2 Univariable Cox regression analysis of mortality-associated factors

In univariable Cox models, lower albumin, prealbumin, hemoglobin, platelet count, PNI, CPNI/1,000, and PANI/1,000 were associated with both mortality outcomes (Table 2). Because the nutritional indices were measured on different scales, the magnitudes of their hazard ratios were not interpreted as direct measures of comparative prognostic importance. APR was reported per 0.1-unit increase to improve interpretability.

**Table 2:** Univariable Cox regression analyses of mortality.

Variable	In-hospital mortality			7-day in-hospital mortality		
	HR (95% CI)	*p* value	*N*/events	HR (95% CI)	*p* value	*N*/events
Sex (female vs. male)	1.1045 (0.6786–1.7977)	0.689	2,221/78	0.9714 (0.5126–1.8406)	0.929	2,221/47
Age, per year	1.0097 (0.9960–1.0235)	0.166	2,221/78	1.0234 (1.0058–1.0413)	0.009	2,221/47
Body mass index, per kg/m^2^	0.8919 (0.7912–1.0054)	0.061	1,595/27	0.9125 (0.7680–1.0842)	0.298	1,595/12
Systolic blood pressure, per mmHg	0.9973 (0.9874–1.0074)	0.600	1,607/77	0.9979 (0.9850–1.0111)	0.755	1,607/46
Albumin, per g/L	0.8641 (0.8466–0.8820)	<0.001	2,221/78	0.8680 (0.8465–0.8901)	<0.001	2,221/47
Prealbumin, per mg/L	0.9857 (0.9821–0.9892)	<0.001	2,221/78	0.9866 (0.9821–0.9911)	<0.001	2,221/47
Hemoglobin, per g/L	0.9792 (0.9742–0.9842)	<0.001	2,221/78	0.9768 (0.9706–0.9831)	<0.001	2,221/47
Lymphocyte count, per 10^9^/L	1.2823 (1.1388–1.4439)	<0.001	2,221/78	1.1612 (0.8927–1.5105)	0.265	2,221/47
Platelet count, per 10^9^/L	0.9876 (0.9835–0.9916)	<0.001	2,221/78	0.9802 (0.9749–0.9856)	<0.001	2,221/47
Alanine aminotransferase, per U/L	1.0011 (0.9973–1.0048)	0.570	2,221/78	1.0004 (0.9939–1.0070)	0.902	2,221/47
Aspartate aminotransferase, per U/L	1.0032 (1.0010–1.0054)	0.004	2,221/78	1.0031 (1.0000–1.0062)	0.049	2,221/47
Creatinine, per μmol/L	1.0035 (1.0009–1.0060)	0.008	2,221/78	1.0034 (1.0001–1.0067)	0.046	2,221/47
Urea nitrogen, per mmol/L	1.0768 (0.9837–1.1788)	0.109	2,221/78	1.1138 (1.0161–1.2209)	0.021	2,221/47
INR, per unit	4.9919 (3.6814–6.7690)	<0.001	2,151/77	5.6971 (3.8987–8.3251)	<0.001	2,151/46
PNI, per unit	0.8844 (0.8663–0.9029)	<0.001	2,221/78	0.8801 (0.8580–0.9028)	<0.001	2,221/47
CPNI/1,000, per unit	0.9116 (0.8695–0.9557)	<0.001	2,221/78	0.8543 (0.7964–0.9165)	<0.001	2,221/47
HALP, per unit	1.0038 (0.9961–1.0115)	0.333	2,221/78	1.0054 (0.9979–1.0130)	0.160	2,221/47
APR, per 0.1-unit increase	0.4586 (0.2764–0.7610)	0.003	2,221/78	0.3930 (0.1974–0.7826)	0.008	2,221/47
PANI/1,000, per unit	0.6203 (0.5680–0.6774)	<0.001	2,221/78	0.6401 (0.5753–0.7122)	<0.001	2,221/47
Other injury (yes vs. no)	1.6549 (0.9609–2.8501)	0.069	2,221/78	1.2627 (0.5656–2.8190)	0.569	2,221/47
Diabetes (yes vs. no)	1.3467 (0.5439–3.3347)	0.520	2,221/78	1.7717 (0.6360–4.9358)	0.274	2,221/47
Hypertension (yes vs. no)	0.5232 (0.2109–1.2976)	0.162	2,221/78	0.9045 (0.3579–2.2861)	0.832	2,221/47
Tracheotomy (yes vs. no)	0.6558 (0.3015–1.4263)	0.287	2,221/78	0.7938 (0.3141–2.0065)	0.626	2,221/47
Mechanical ventilation (yes vs. no)	1.5939 (0.7659–3.3170)	0.212	2,221/78	1.5579 (0.6164–3.9375)	0.349	2,221/47
Craniotomy/brain surgery (yes vs. no)	1.1133 (0.6842–1.8114)	0.666	2,221/78	1.1470 (0.6138–2.1431)	0.667	2,221/47
IVC filter (yes vs. no)	2.3687 (0.7460–7.5212)	0.144	2,221/78	2.3077 (0.5598–9.5130)	0.247	2,221/47
Orthopedic surgery (yes vs. no)	0.3906 (0.0543–2.8104)	0.350	2,221/78	NE	NE	2,221/47

Each nutritional index is reported once on an interpretable scale.

### 3.3 Comparative discriminative performance of nutritional indices

**Figure 2:**
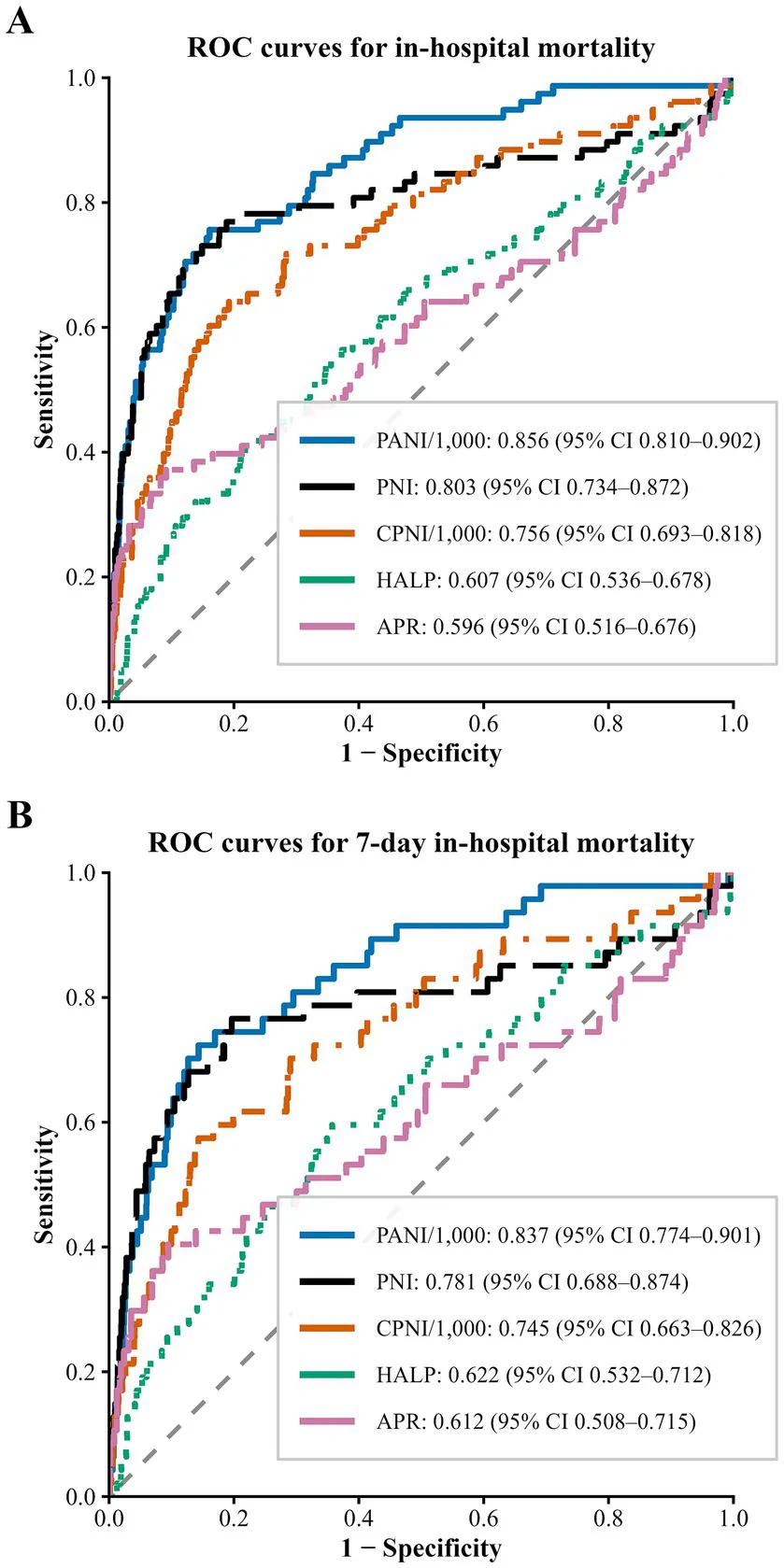
Receiver operating characteristic curves of the five nutritional indices for in-hospital mortality **(A)** and 7-day in-hospital mortality **(B)**. AUCs and 95% confidence intervals were calculated using DeLong’s method. AUC, area under the receiver operating characteristic curve; APR, Albumin-to-prealbumin ratio; CPNI, Composite prealbumin nutritional index; HALP, Hemoglobin-albumin-lymphocyte-platelet score; PANI, Prealbumin–albumin nutritional index; PNI, Prognostic nutritional index.

PANI/1,000 had the highest observed AUC among the five indices for in-hospital mortality (0.856; 95% CI, 0.810–0.902) and 7-day in-hospital mortality (0.837; 95% CI, 0.774–0.901) (Table 3, Figure 2). Compared with PNI, the AUC difference was statistically significant for in-hospital mortality (*p* = 0.020) but not for 7-day in-hospital mortality (*p* = 0.082). Accordingly, the results support better observed discrimination for in-hospital mortality but do not establish general superiority across both outcomes.

**Table 3:** Discrimination of the five nutritional indices for mortality.

Index	In-hospital mortality				7-day in-hospital mortality			
	AUC (95% CI)	Cutoff	Sensitivity	Specificity	AUC (95% CI)	Cutoff	Sensitivity	Specificity
PNI	0.803 (0.734–0.872)	39.175	0.731	0.850	0.781 (0.688–0.874)	40.525	0.766	0.801
CPNI/1,000	0.756 (0.693–0.818)	5.1778	0.641	0.808	0.745 (0.663–0.826)	4.2592	0.574	0.857
HALP	0.607 (0.536–0.678)	24.597	0.551	0.644	0.622 (0.532–0.712)	24.597	0.596	0.643
APR	0.596 (0.516–0.676)	0.1217	0.372	0.909	0.612 (0.508–0.715)	0.1217	0.404	0.905
PANI/1,000	0.856 (0.810–0.902)	6.0060	0.756	0.839	0.837 (0.774–0.901)	5.7019	0.723	0.856

Cutoffs are reported on the analysis scale shown for each index; PANI and CPNI values are divided by 1,000.

**Figure 3:**
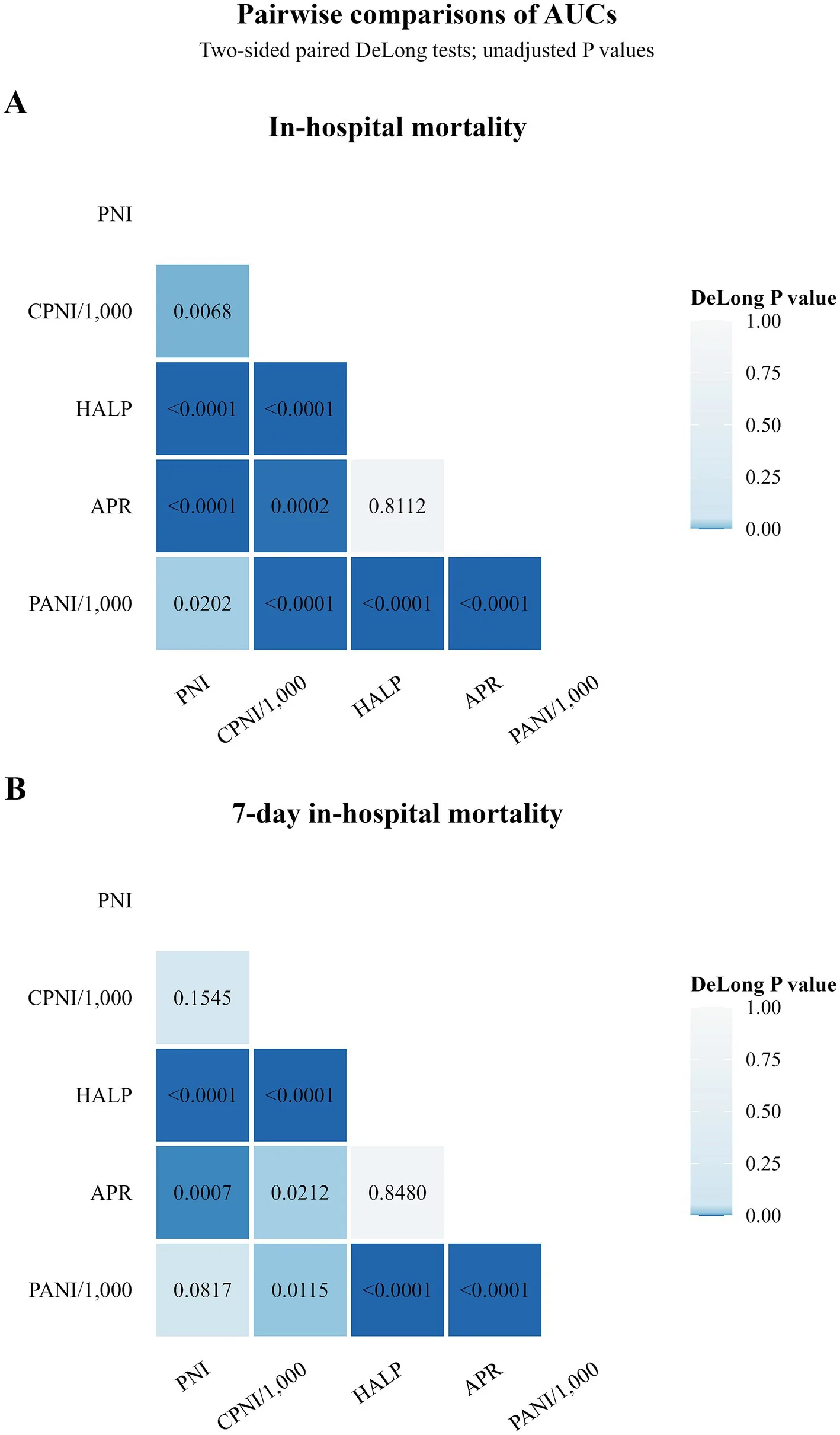
Pairwise comparisons of AUCs for the five nutritional indices for in-hospital mortality **(A)** and 7-day in-hospital mortality **(B)**. Cells in the lower triangle show unadjusted two-sided *p* values from paired DeLong tests; the diagonal and upper triangle are intentionally blank. Because multiple pairwise comparisons were performed without multiplicity adjustment, the results were considered exploratory. AUC, area under the receiver operating characteristic curve.

Pairwise DeLong comparisons further showed that PANI/1,000 had greater observed discrimination than several other indices. However, its advantage over PNI was statistically significant only for in-hospital mortality and not for 7-day in-hospital mortality (Figure 3). Therefore, these findings do not establish general superiority of PANI over PNI across both outcomes.

### 3.4 Multivariable cox regression analysis of PANI and mortality risk

**Figure 4:**
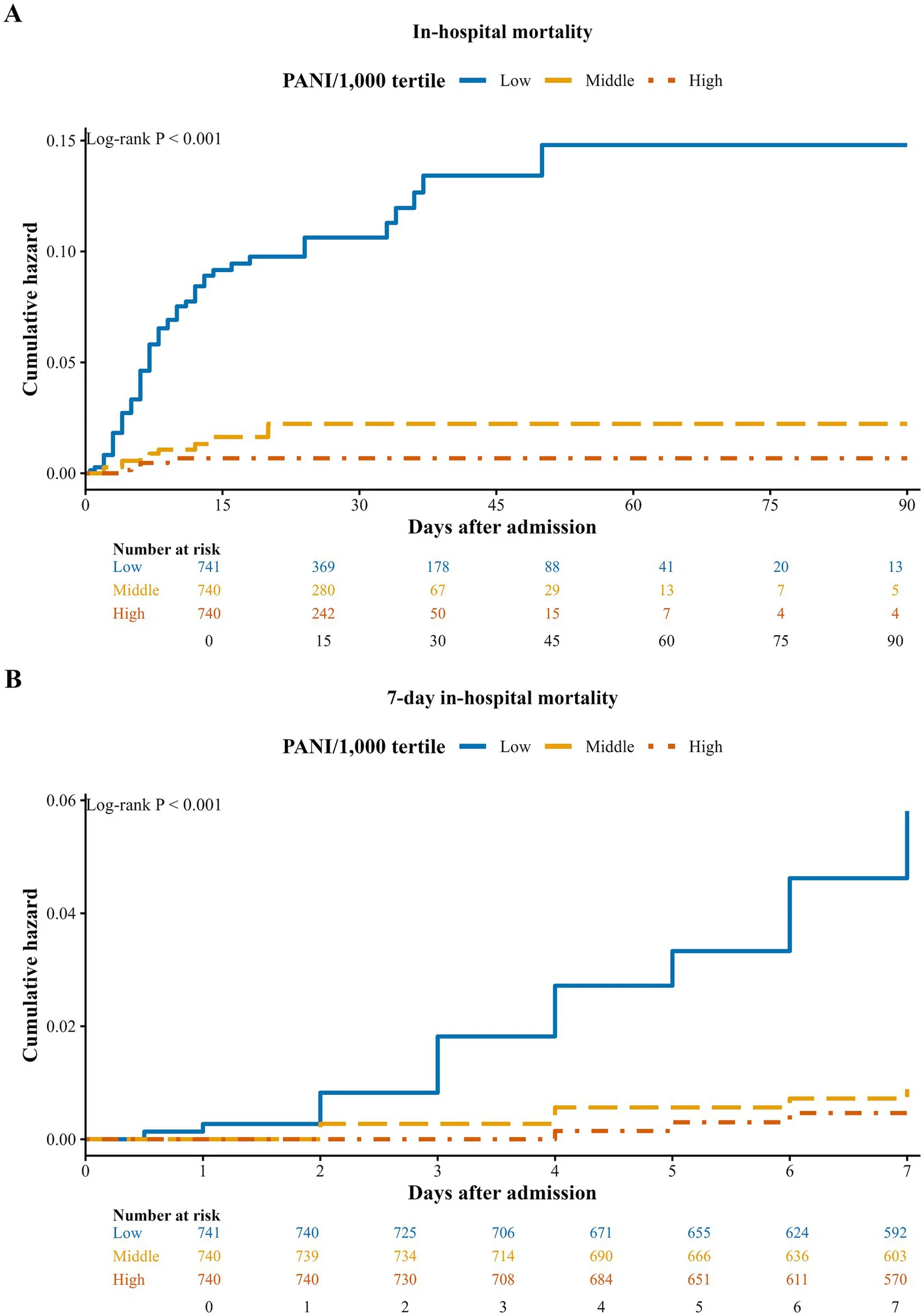
Cumulative hazard of mortality according to tertiles of PANI/1,000. **(A)** In-hospital mortality displayed through 90 days. **(B)** 7-day in-hospital mortality displayed through day 7. Curves were derived from Kaplan–Meier survival estimates, and between-group *p* values were obtained from log-rank tests. The log-rank test for in-hospital mortality used the complete available follow-up; the 90-day restriction applies only to visualization. Numbers at risk are shown below each panel. PANI, prealbumin–albumin nutritional index.

To examine the adjusted association of PANI with mortality, we fitted Models 1–3, with Model 3 serving as the primary adjusted model, together with a supplementary extended baseline model (Model 4) (Table 4), and plotted outcome curves by PANI/1,000 tertile (Figure 4).

**Table 4:** Association between PANI/1,000 and mortality in Cox regression models.

Section	Outcome	Exposure	Model	HR (95% CI)	*p* value	*N*/events
A. Continuous PANI/1,000	In-hospital mortality	PANI/1,000, per 1-unit increase	Model 1	0.6203 (0.5680–0.6774)	<0.001	2,221/78
		PANI/1,000, per 1-unit increase	Model 2	0.6168 (0.5647–0.6738)	<0.001	2,221/78
		PANI/1,000, per 1-unit increase	Model 3	0.6128 (0.5601–0.6705)	<0.001	2,221/78
		PANI/1,000, per 1-unit increase	Model 4	0.6532 (0.5928–0.7196)	<0.001	2,151/77
	7-day in-hospital mortality	PANI/1,000, per 1-unit increase	Model 1	0.6401 (0.5753–0.7122)	<0.001	2,221/47
		PANI/1,000, per 1-unit increase	Model 2	0.6433 (0.5778–0.7163)	<0.001	2,221/47
		PANI/1,000, per 1-unit increase	Model 3	0.6402 (0.5741–0.7139)	<0.001	2,221/47
		PANI/1,000, per 1-unit increase	Model 4	0.6900 (0.6162–0.7727)	<0.001	2,151/46
B. PANI/1,000 tertiles	In-hospital mortality	Low tertile (reference)	Model 1	1.000 (Reference)	–	2,221/78
		Middle tertile vs. Low	Model 1	0.1914 (0.1006–0.3639)	<0.001	2,221/78
		High tertile vs. Low	Model 1	0.0747 (0.0271–0.2058)	<0.001	2,221/78
		Low tertile (reference)	Model 2	1.000 (Reference)	–	2,221/78
		Middle tertile vs. Low	Model 2	0.1848 (0.0968–0.3528)	<0.001	2,221/78
		High tertile vs. Low	Model 2	0.0679 (0.0242–0.1901)	<0.001	2,221/78
		Low tertile (reference)	Model 3	1.000 (Reference)	–	2,221/78
		Middle tertile vs. Low	Model 3	0.1861 (0.0975–0.3552)	<0.001	2,221/78
		High tertile vs. Low	Model 3	0.0708 (0.0253–0.1985)	<0.001	2,221/78
		Low tertile (reference)	Model 4	1.000 (Reference)	–	2,151/77
		Middle tertile vs. Low	Model 4	0.2466 (0.1275–0.4768)	<0.001	2,151/77
		High tertile vs. Low	Model 4	0.0992 (0.0351–0.2805)	<0.001	2,151/77
	7-day in-hospital mortality	Low tertile (reference)	Model 1	1.000 (Reference)	–	2,221/47
		Middle tertile vs. Low	Model 1	0.1546 (0.0654–0.3658)	<0.001	2,221/47
		High tertile vs. Low	Model 1	0.0792 (0.0244–0.2565)	<0.001	2,221/47
		Low tertile (reference)	Model 2	1.000 (Reference)	–	2,221/47
		Middle tertile vs. Low	Model 2	0.1573 (0.0660–0.3748)	<0.001	2,221/47
		High tertile vs. Low	Model 2	0.0795 (0.0238–0.2651)	<0.001	2,221/47
		Low tertile (reference)	Model 3	1.000 (Reference)	–	2,221/47
		Middle tertile vs. Low	Model 3	0.1594 (0.0669–0.3799)	<0.001	2,221/47
		High tertile vs. Low	Model 3	0.0822 (0.0246–0.2746)	<0.001	2,221/47
		Low tertile (reference)	Model 4	1.000 (Reference)	–	2,151/46
		Middle tertile vs. Low	Model 4	0.2109 (0.0870–0.5114)	<0.001	2,151/46
		High tertile vs. Low	Model 4	0.1130 (0.0334–0.3830)	<0.001	2,151/46

Model 3 was the primary adjusted model, and Model 4 was the supplementary extended baseline model.

In the primary adjusted model (Model 3), each 1-unit increase in PANI/1,000 was associated with lower hazards of in-hospital mortality (HR, 0.613; 95% CI, 0.560–0.671; *p* < 0.001) and 7-day in-hospital mortality (HR, 0.640; 95% CI, 0.574–0.714; p < 0.001).

In the supplementary extended baseline model (Model 4), each 1-unit increase in PANI/1,000 remained associated with lower hazards of in-hospital mortality (HR, 0.653; 95% CI, 0.593–0.720; *p* < 0.001) and 7-day in-hospital mortality (HR, 0.690; 95% CI, 0.616–0.773; *p* < 0.001). A complete-case sensitivity analysis additionally incorporating body mass index and systolic blood pressure showed directionally consistent inverse associations with in-hospital mortality (HR, 0.562; 95% CI, 0.466–0.678; *p* < 0.001) and 7-day in-hospital mortality (HR, 0.677; 95% CI, 0.528–0.870; *p* = 0.002) (Supplementary Table S5).

Outcome curves showed more events in the lowest PANI/1,000 tertile. Cumulative hazards differed significantly across PANI/1,000 tertiles for both outcomes (both log-rank *p* < 0.001) (Figure 4). Numbers at risk are presented below each panel, and time is reported in days.

### 3.5 Exploratory spline and robustness analyses

In exploratory three-knot restricted cubic spline analyses adjusted using the Model 3 covariate set, the overall associations between PANI/1,000 and both mortality outcomes were statistically significant (both *P* for overall association < 0.001). The tests for nonlinearity were nominally significant for in-hospital mortality (*P* for nonlinearity = 0.008) and 7-day in-hospital mortality (*P* for nonlinearity = 0.018). However, the nonlinear signal was highly sensitive to a single upper-tail death event with the highest PANI value among deaths. After exclusion of this observation, the corresponding tests for nonlinearity were no longer significant (*p* = 0.453 and *p* = 0.846, respectively) (Supplementary Figure S4, Supplementary Table S7).

Importantly, exclusion of this influential observation did not materially alter the overall inverse association in the primary adjusted Cox model. The corresponding HRs per 1-unit increase in PANI/1,000 were 0.594 (95% CI, 0.542–0.652; *p* < 0.001) for in-hospital mortality and 0.612 (95% CI, 0.546–0.686; *p* < 0.001) for 7-day in-hospital mortality. Therefore, the apparent spline curvature was considered model-sensitive and was not interpreted as evidence of a robust biological nonlinear relationship or a clinically meaningful threshold.

Pairwise Spearman correlation coefficients among the five nutritional indices ranged from −0.55 to 0.81, and variance inflation factors ranged from 1.68 to 4.83; no variance inflation factor exceeded 5 (Supplementary Table S1). The proportional hazards assumption was not rejected. For in-hospital mortality, the global *p* values were 0.427 for Model 3 and 0.308 for Model 4; for 7-day in-hospital mortality, the corresponding global *p* values were 0.385 and 0.750 (Supplementary Table S2).

Formal interaction tests are presented in Supplementary Figure S2, Supplementary Table S3; corresponding BH-FDR-adjusted *p* values are reported in Supplementary Table S3. For in-hospital mortality, nominal interaction signals were observed for albumin (*P* for interaction = 0.004; FDR-adjusted *p* = 0.045), hypertension (*p* = 0.023; FDR-adjusted *p* = 0.093), and tracheotomy (*p* = 0.015; FDR-adjusted *p* = 0.088). For 7-day in-hospital mortality, nominal interaction signals were observed for albumin (*p* = 0.015; FDR-adjusted *p* = 0.089), diabetes (*p* = 0.025; FDR-adjusted *p* = 0.101), hypertension (*p* = 0.035; FDR-adjusted *p* = 0.104), and tracheotomy (*p* = 0.009; FDR-adjusted *p* = 0.089). Thus, none of the clinically interpretable interaction signals remained statistically significant after correction for multiple testing. Although the PANI-by-albumin interaction for in-hospital mortality remained below 0.05 after FDR correction, albumin is a direct multiplicative component of PANI; this finding therefore reflects structural mathematical coupling and was not interpreted as biological effect modification.

Bootstrap internal validation, calibration assessment, and decision-curve analyses are presented in Supplementary Figure S3, Supplementary Table S4. For the baseline covariate model plus PANI, the optimism-corrected AUCs were 0.858 for in-hospital mortality and 0.833 for 7-day in-hospital mortality. The corresponding optimism-corrected Brier scores were 0.029 and 0.020, the corrected calibration intercepts were −0.011 and −0.001, and the corrected calibration slopes were 0.915 and 0.888, respectively. The Hosmer–Lemeshow *p* values were 0.476 and 0.305, respectively. Across most examined threshold probabilities, decision-curve analysis showed slightly greater net benefit for the baseline covariate model plus PANI than for the baseline covariate model alone; however, the incremental improvement was modest.

## 4 Discussion

In this single-center retrospective cohort, lower admission PANI was associated with higher in-hospital and 7-day in-hospital mortality. PANI showed higher observed discrimination than the other evaluated indices, although its advantage over PNI was statistically supported only for in-hospital mortality. The findings suggest that PANI may be a useful candidate marker for further study; they do not establish that PANI improves clinical decision-making or outperforms validated TBI prognostic models.

Previous studies have associated albumin, prealbumin, and PNI with outcomes after TBI (12–14). PANI combines two correlated negative acute-phase proteins and may summarize changes related to nutritional reserve, inflammation, fluid shifts, hepatic synthesis, and overall illness severity. Its relatively high AUC may partly reflect the strong association of both components with acute physiologic disturbance. The observed comparison with PNI was favorable for in-hospital mortality, but the difference for 7-day in-hospital mortality was inconclusive. Comparisons with HALP and APR should also be interpreted in light of their different scales, component variability, and the absence of external validation. HALP has also been associated with mortality in neurocritical care populations, although evidence specific to TBI remains limited (17). The five indices represent overlapping but distinct biological constructs. PNI combines albumin with lymphocyte count and therefore incorporates both protein-related and immune components. CPNI additionally includes prealbumin in a multiplicative formulation, which may increase both biological integration and numerical variability. HALP may be influenced by traumatic bleeding, hemodilution, blood transfusion, platelet consumption, and reactive hematologic changes. APR may become unstable when prealbumin concentrations are low. PANI has a simpler two-component structure, although both albumin and prealbumin remain sensitive to inflammation, fluid shifts, hepatic synthesis, and acute illness severity. Although both three- and four-knot spline models produced nominal evidence of nonlinearity in the full cohort, the nonlinear signal was highly sensitive to a single upper-tail death event. After exclusion of this observation, the tests for nonlinearity were no longer significant, whereas the overall inverse association between PANI and mortality remained statistically significant. We therefore do not interpret the observed curvature as robust evidence of a biological nonlinear relationship or a clinically meaningful threshold. The principal finding of this study is the overall inverse association between PANI and both short-term mortality outcomes rather than a nonlinear or threshold relationship.

The exploratory subgroup analyses also require cautious interpretation. The apparent interaction between PANI and albumin should not be interpreted as biological effect modification because albumin is a direct multiplicative component of PANI (PANI = albumin × prealbumin), creating structural mathematical dependence. Nominal differences according to hypertension or diabetes could conceivably reflect differences in vascular or metabolic reserve, whereas tracheotomy may primarily reflect injury severity, prolonged airway management, or treatment selection rather than true biological effect modification. These explanations remain speculative. After correction for multiple testing, none of the clinically interpretable interaction signals remained statistically significant. These subgroup findings were not externally validated and should not be used as a basis for clinical decision-making.

PANI can be calculated when both albumin and prealbumin measurements are available. Nevertheless, the current study does not show that PANI adds information beyond Glasgow Coma Scale score, imaging findings, pupillary response, or established IMPACT and CRASH models. It also does not demonstrate that PANI-guided nutritional or monitoring interventions improve outcomes. Its potential clinical role therefore remains uncertain pending comparison with established TBI prognostic models and external validation.

The supplementary prediction analyses provided additional context for the potential clinical utility of PANI. The model combining PANI with the available baseline covariates showed calibration intercepts close to zero and calibration slopes below but close to one after bootstrap correction. Nevertheless, improvements in optimism-corrected discrimination and decision-curve net benefit over the baseline covariate model were modest. These findings support further external validation rather than immediate clinical implementation.

Several mechanisms may contribute to lower albumin and prealbumin concentrations after TBI, including the acute-phase response, altered hepatic protein synthesis, capillary leakage, hemodilution, and pre-existing nutritional vulnerability (18–27). Hematologic components of PNI, CPNI, and HALP may also vary with stress responses, bleeding, fluid resuscitation, transfusion, and coagulopathy (28–32). These mechanisms are biologically plausible but were not measured directly in this study; therefore, they should be presented as possible explanations rather than evidence that PANI specifically measures malnutrition.

Strengths include the consecutive single-center cohort, use of clinically available laboratory measurements, assessment of two short-term mortality outcomes, and direct comparison with several composite indices. The consistent association across adjustment models also supports the robustness of the observed statistical association within this dataset.

This study has important limitations. First, the retrospective single-center design and complete-case selection may introduce selection bias and limit generalizability. Prealbumin measurements were unavailable in 290 patients (11.5%) in the source cohort. Patients without available prealbumin measurements had substantially higher observed in-hospital and 7-day in-hospital mortality than those with available measurements, indicating that prealbumin missingness was unlikely to be completely random. The reasons for individual prealbumin test ordering could not be retrospectively determined. Consequently, complete-case inclusion may have introduced selection bias; however, the direction and magnitude of this bias on the estimated PANI–mortality association cannot be determined from the present data. Second, key determinants of TBI prognosis, including Glasgow Coma Scale score, pupillary reactivity, computed tomography findings, hypotension, hypoxia, and extracranial injury severity, were unavailable; substantial residual confounding by injury severity is therefore likely. Third, several adjusted treatment variables occurred after admission and may represent mediators or markers of severity. Fourth, the exact timing of laboratory sampling and its temporal relationships with injury, resuscitation, blood transfusion, albumin administration, and surgery could not be retrospectively determined. Information on preadmission anticoagulant or antiplatelet therapy and documented histories of chronic liver or kidney disease was also unavailable. The 7-day outcome captured only in-hospital deaths because post-discharge mortality was unavailable. Fifth, PANI was developed, its data-derived cutoff was selected, and its performance was evaluated in the same cohort. Although bootstrap internal validation, calibration assessment, and decision-curve analysis were performed, external validation was unavailable. Incremental value beyond established TBI prognostic models could not be evaluated because major neurological and imaging severity variables were unavailable. Sixth, only 78 in-hospital and 47 7-day in-hospital deaths occurred, limiting the stability of complex and subgroup analyses. The apparent spline nonlinearity was highly sensitive to a single upper-tail death event, underscoring the instability of flexible exposure-response modeling in regions with sparse outcome events. Finally, baseline concentrations alone cannot distinguish nutritional status from inflammation, fluid shifts, hepatic dysfunction, or acute illness severity.

## 5 Conclusion

Lower admission PANI was associated with higher in-hospital and 7-day in-hospital mortality in hospitalized patients with TBI. However, the single-center design, potential selection bias, residual confounding, and absence of key neurological and imaging severity variables preclude conclusions regarding its incremental clinical value. The potential clinical value of PANI can only be evaluated after validation in multicenter prospective studies incorporating complete neurological and imaging assessments.

## Data Availability

The original contributions presented in the study are included in the article/Supplementary material, further inquiries can be directed to the corresponding author.

## Supplementary material

SUPPLEMENTARY FIGURE S1 Compositional architecture of the five nutritional indices. Solid arrows indicate numerator or additive components, and dashed arrows indicate denominator components. ALB, albumin; APR, albumin-to-prealbumin ratio; CPNI, composite prealbumin nutritional index; HALP, hemoglobin–albumin–lymphocyte–platelet score; Hb, hemoglobin; L, lymphocyte count; PANI, prealbumin–albumin nutritional index; PLT, platelet count; PNI, prognostic nutritional index; PreALB, prealbumin.

SUPPLEMENTARY FIGURE S2 **(A)** Exploratory subgroup analyses of the association between PANI/1,000 and mortality. In-hospital mortality. **(B)** 7-day in-hospital mortality. Points and horizontal lines show adjusted hazard ratios and 95% confidence intervals per 1-unit increase in PANI/1,000. *p* values for interaction were obtained from likelihood-ratio tests of multiplicative interaction terms. The interaction *p* values displayed in the figure are unadjusted; corresponding Benjamini–Hochberg false discovery rate-adjusted *p* values are reported in Supplementary Table S3. The vertical dashed line indicates HR = 1. NE indicates that a stable estimate could not be reported because of sparse events. Subgroup analyses were exploratory.

SUPPLEMENTARY FIGURE S3 Bootstrap-corrected calibration and decision-curve analyses. **(A, B)** Calibration plots for the baseline covariate model plus PANI for in-hospital mortality and 7-day in-hospital mortality, respectively. Blue lines show apparent calibration and red dashed lines show bootstrap-corrected calibration; the gray dashed line represents perfect calibration. **(C, D)** Decision-curve analyses for in-hospital mortality and 7-day in-hospital mortality, respectively, comparing the baseline covariate model, baseline covariate model plus PANI, PANI-only model, treat-all strategy, and treat-none strategy. Prediction analyses used binary logistic regression and were supplementary to the primary time-to-event Cox analyses.

SUPPLEMENTARY FIGURE S4 Sensitivity analyses of the association between PANI/1,000 and mortality using three-knot restricted cubic splines. Restricted cubic spline models were adjusted using the Model 3 covariate set (age, sex, diabetes, hypertension, tracheotomy, mechanical ventilation, craniotomy or brain surgery, and inferior vena cava filter placement). Knots were placed at the 10th, 50th, and 90th percentiles of PANI/1,000. Rug marks along the *x*-axis show the observed PANI/1,000 distribution in each analysis dataset. Panels A and B show the full-cohort analyses, whereas Panels C and D show influence analyses after exclusion of the single upper-tail death event with the highest PANI value. The same knot locations were retained in the influence analyses. The loss of statistical evidence for nonlinearity after exclusion of this observation indicates that the apparent nonlinear signal was not robust.
