# Supplementary material for: Association of the prealbumin–albumin nutritional index with short-term mortality in hospitalized patients with traumatic brain injury
Source: Front Nutr. 2026 Sep 3;13:1910413. doi: 10.3389/fnut.2026.1910413 (PMC13581652; doi:10.3389/fnut.2026.1910413)
Supplement: Supplementary file 1 [file Table_1.DOCX]

**Table S1.** Mathematical correlation and collinearity among the five nutritional indices. The indices were assessed for collinearity but were entered into prognostic models separately.

**A. Spearman correlation matrix**

| **Index** | **PNI** | **CPNI/1,000** | **HALP** | **APR** | **PANI/1,000** |
| --- | --- | --- | --- | --- | --- |
| PNI | 1.00 | 0.81 | 0.62 | 0.01 | 0.67 |
| CPNI/1,000 | 0.81 | 1.00 | 0.72 | -0.33 | 0.69 |
| HALP | 0.62 | 0.72 | 1.00 | 0.00 | 0.31 |
| APR | 0.01 | -0.33 | 0.00 | 1.00 | -0.55 |
| PANI/1,000 | 0.67 | 0.69 | 0.31 | -0.55 | 1.00 |

**B. Variance inflation factors**

| **Index** | **R²** | **VIF** | **Tolerance** |
| --- | --- | --- | --- |
| PNI | 0.793 | 4.83 | 0.207 |
| CPNI/1,000 | 0.711 | 3.46 | 0.289 |
| HALP | 0.405 | 1.68 | 0.595 |
| APR | 0.563 | 2.29 | 0.437 |
| PANI/1,000 | 0.705 | 3.39 | 0.295 |

Abbreviations: APR, albumin-to-prealbumin ratio; CPNI, composite prealbumin nutritional index; HALP, hemoglobin–albumin–lymphocyte–platelet score; PANI, prealbumin–albumin nutritional index; PNI, prognostic nutritional index; VIF, variance inflation factor.
