# Supplementary material for: Association of the prealbumin–albumin nutritional index with short-term mortality in hospitalized patients with traumatic brain injury
Source: Front Nutr. 2026 Sep 3;13:1910413. doi: 10.3389/fnut.2026.1910413 (PMC13581652; doi:10.3389/fnut.2026.1910413)
Supplement: Supplementary file 2 [file Table_2.DOCX]

**Table S2.** Tests of the proportional hazards assumption. Scaled Schoenfeld residuals were assessed for Models 3 and 4.

| **Outcome** | **Model** | **Term** | **Chi-square** | **df** | **P value** | **N/events** |
| --- | --- | --- | --- | --- | --- | --- |
|  |  |  |  |  |  |  |
| In-hospital mortality | Model 3 | PANI/1,000 | 0.003 | 1 | 0.959 | 2221/78 |
|  |  | Age | 2.190 | 1 | 0.139 | 2221/78 |
|  |  | Sex | 0.748 | 1 | 0.387 | 2221/78 |
|  |  | Diabetes | 0.012 | 1 | 0.913 | 2221/78 |
|  |  | Hypertension | 3.424 | 1 | 0.064 | 2221/78 |
|  |  | Tracheotomy | 0.788 | 1 | 0.375 | 2221/78 |
|  |  | Mechanical ventilation | 0.320 | 1 | 0.571 | 2221/78 |
|  |  | Craniotomy/brain surgery | 0.422 | 1 | 0.516 | 2221/78 |
|  |  | Inferior vena cava filter | 0.410 | 1 | 0.522 | 2221/78 |
|  |  | Global test | 9.108 | 9 | 0.427 | 2221/78 |
|  | Model 4 | PANI/1,000 | 0.023 | 1 | 0.880 | 2151/77 |
|  |  | Age | 2.021 | 1 | 0.155 | 2151/77 |
|  |  | Sex | 0.492 | 1 | 0.483 | 2151/77 |
|  |  | Diabetes | 0.044 | 1 | 0.834 | 2151/77 |
|  |  | Hypertension | 3.152 | 1 | 0.076 | 2151/77 |
|  |  | Other injury | 0.863 | 1 | 0.353 | 2151/77 |
|  |  | Alanine aminotransferase | 0.134 | 1 | 0.714 | 2151/77 |
|  |  | Creatinine | 0.523 | 1 | 0.470 | 2151/77 |
|  |  | International normalized ratio | 1.337 | 1 | 0.248 | 2151/77 |
|  |  | Global test | 10.552 | 9 | 0.308 | 2151/77 |
| 7-day in-hospital mortality | Model 3 | PANI/1,000 | 2.563 | 1 | 0.109 | 2221/47 |
|  |  | Age | 0.169 | 1 | 0.681 | 2221/47 |
|  |  | Sex | 0.833 | 1 | 0.361 | 2221/47 |
|  |  | Diabetes | 0.964 | 1 | 0.326 | 2221/47 |
|  |  | Hypertension | 0.340 | 1 | 0.560 | 2221/47 |
|  |  | Tracheotomy | 0.229 | 1 | 0.632 | 2221/47 |
|  |  | Mechanical ventilation | 1.155 | 1 | 0.282 | 2221/47 |
|  |  | Craniotomy/brain surgery | 0.182 | 1 | 0.670 | 2221/47 |
|  |  | Inferior vena cava filter | 2.001 | 1 | 0.157 | 2221/47 |
|  |  | Global test | 9.584 | 9 | 0.385 | 2221/47 |
|  | Model 4 | PANI/1,000 | 1.687 | 1 | 0.194 | 2151/46 |
|  |  | Age | 0.004 | 1 | 0.950 | 2151/46 |
|  |  | Sex | 1.323 | 1 | 0.250 | 2151/46 |
|  |  | Diabetes | 1.051 | 1 | 0.305 | 2151/46 |
|  |  | Hypertension | 0.425 | 1 | 0.515 | 2151/46 |
|  |  | Other injury | 0.019 | 1 | 0.891 | 2151/46 |
|  |  | Alanine aminotransferase | 0.263 | 1 | 0.608 | 2151/46 |
|  |  | Creatinine | 0.104 | 1 | 0.747 | 2151/46 |
|  |  | International normalized ratio | 0.692 | 1 | 0.405 | 2151/46 |
|  |  | Global test | 5.903 | 9 | 0.750 | 2151/46 |

The chi-square test was obtained from survival::cox.zph using the Kaplan–Meier time transformation. A P value below 0.05 would indicate evidence against the proportional hazards assumption.

Model 3 adjusted for age, sex, diabetes, hypertension, tracheotomy, mechanical ventilation, craniotomy or brain surgery, and inferior vena cava filter placement. Model 4 adjusted for age, sex, diabetes, hypertension, other injury, alanine aminotransferase, creatinine, and international normalized ratio.

Abbreviation: PANI, prealbumin–albumin nutritional index.
