# Supplementary material for: Association of the prealbumin–albumin nutritional index with short-term mortality in hospitalized patients with traumatic brain injury
Source: Front Nutr. 2026 Sep 3;13:1910413. doi: 10.3389/fnut.2026.1910413 (PMC13581652; doi:10.3389/fnut.2026.1910413)
Supplement: Supplementary file 3 [file Table_3.DOCX]

**Table S3.** Exploratory subgroup analyses of the association between PANI/1,000 and mortality.

| **Outcome** | **Subgroup** | **Level** | **N** | **Events** | **HR (95% CI)** | **P value** | **P for interaction** | **FDR-adjusted P** |
| --- | --- | --- | --- | --- | --- | --- | --- | --- |
| In-hospital mortality | Age | ≤50 | 1065 | 30 | 0.555 (0.470–0.655) | <0.001 | 0.120 | 0.205 |
|  |  | ≥51 | 1156 | 48 | 0.654 (0.584–0.733) | <0.001 |  |  |
|  | Sex | Male | 1590 | 55 | 0.633 (0.572–0.701) | <0.001 | 0.095 | 0.189 |
|  |  | Female | 631 | 23 | 0.551 (0.452–0.671) | <0.001 |  |  |
|  | Albumin | ≤39.7 | 1123 | 66 | 0.562 (0.498–0.634) | <0.001 | 0.004 | 0.045 |
|  |  | ≥39.8 | 1098 | 12 | 0.895 (0.707–1.132) | 0.354 |  |  |
|  | Hemoglobin | ≤128.9 | 1088 | 64 | 0.590 (0.525–0.663) | <0.001 | 0.073 | 0.189 |
|  |  | ≥129.0 | 1133 | 14 | 0.709 (0.583–0.863) | <0.001 |  |  |
|  | Lymphocyte count | ≤1.1 | 1160 | 36 | 0.616 (0.534–0.709) | <0.001 | 0.766 | 0.835 |
|  |  | ≥1.2 | 1061 | 42 | 0.604 (0.538–0.678) | <0.001 |  |  |
|  | Platelet count | ≤179 | 1110 | 63 | 0.611 (0.548–0.680) | <0.001 | 0.278 | 0.370 |
|  |  | ≥180 | 1111 | 15 | 0.680 (0.558–0.829) | <0.001 |  |  |
|  | Other injury | No | 1961 | 61 | 0.617 (0.559–0.680) | <0.001 | 0.868 | 0.868 |
|  |  | Yes | 260 | 17 | 0.570 (0.439–0.740) | <0.001 |  |  |
|  | Diabetes | No | 2111 | 73 | 0.599 (0.545–0.659) | <0.001 | 0.094 | 0.189 |
|  |  | Yes | 110 | 5 | 0.824 (0.577–1.176) | 0.286 |  |  |
|  | Hypertension | No | 1961 | 73 | 0.593 (0.539–0.653) | <0.001 | 0.023 | 0.093 |
|  |  | Yes | 260 | 5 | 0.822 (0.579–1.167) | 0.273 |  |  |
|  | Tracheotomy | No | 1935 | 71 | 0.629 (0.574–0.690) | <0.001 | 0.015 | 0.088 |
|  |  | Yes | 286 | 7 | 0.317 (0.162–0.619) | <0.001 |  |  |
|  | Mechanical ventilation | No | 2059 | 70 | 0.607 (0.552–0.668) | <0.001 | 0.654 | 0.785 |
|  |  | Yes | 162 | 8 | 0.590 (0.418–0.832) | 0.003 |  |  |
|  | Craniotomy/brain surgery | No | 1610 | 55 | 0.634 (0.570–0.704) | <0.001 | 0.169 | 0.253 |
|  |  | Yes | 611 | 23 | 0.562 (0.467–0.676) | <0.001 |  |  |
|  | IVC filter | No | 2179 | 75 | 0.621 (0.567–0.680) | <0.001 | NE | NE |
|  |  | Yes | 42 | 3 | NE | NE |  |  |

| **Outcome** | **Subgroup** | **Level** | **N** | **Events** | **HR (95% CI)** | **P value** | **P for interaction** | **FDR-adjusted P** |
| --- | --- | --- | --- | --- | --- | --- | --- | --- |
| 7-day in-hospital mortality | Age | ≤50 | 1065 | 14 | 0.620 (0.512–0.752) | <0.001 | 0.743 | 0.860 |
|  |  | ≥51 | 1156 | 33 | 0.648 (0.566–0.742) | <0.001 |  |  |
|  | Sex | Male | 1590 | 34 | 0.651 (0.575–0.738) | <0.001 | 0.576 | 0.768 |
|  |  | Female | 631 | 13 | 0.608 (0.482–0.768) | <0.001 |  |  |
|  | Albumin | ≤39.7 | 1123 | 38 | 0.573 (0.492–0.666) | <0.001 | 0.015 | 0.089 |
|  |  | ≥39.8 | 1098 | 9 | 0.896 (0.686–1.171) | 0.421 |  |  |
|  | Hemoglobin | ≤128.9 | 1088 | 37 | 0.622 (0.538–0.718) | <0.001 | 0.176 | 0.303 |
|  |  | ≥129.0 | 1133 | 10 | 0.719 (0.562–0.919) | 0.008 |  |  |
|  | Lymphocyte count | ≤1.1 | 1160 | 24 | 0.666 (0.565–0.783) | <0.001 | 0.396 | 0.594 |
|  |  | ≥1.2 | 1061 | 23 | 0.613 (0.530–0.709) | <0.001 |  |  |
|  | Platelet count | ≤179 | 1110 | 38 | 0.630 (0.553–0.718) | <0.001 | 0.115 | 0.231 |
|  |  | ≥180 | 1111 | 9 | 0.748 (0.585–0.957) | 0.021 |  |  |
|  | Other injury | No | 1961 | 40 | 0.636 (0.566–0.715) | <0.001 | 0.860 | 0.860 |
|  |  | Yes | 260 | 7 | 0.632 (0.451–0.885) | 0.008 |  |  |
|  | Diabetes | No | 2111 | 43 | 0.615 (0.548–0.691) | <0.001 | 0.025 | 0.101 |
|  |  | Yes | 110 | 4 | 0.963 (0.665–1.395) | 0.843 |  |  |
|  | Hypertension | No | 1961 | 42 | 0.614 (0.545–0.691) | <0.001 | 0.035 | 0.104 |
|  |  | Yes | 260 | 5 | 0.822 (0.579–1.167) | 0.273 |  |  |
|  | Tracheotomy | No | 1935 | 42 | 0.665 (0.595–0.744) | <0.001 | 0.009 | 0.089 |
|  |  | Yes | 286 | 5 | 0.233 (0.081–0.669) | 0.007 |  |  |
|  | Mechanical ventilation | No | 2059 | 42 | 0.636 (0.566–0.714) | <0.001 | 0.793 | 0.860 |
|  |  | Yes | 162 | 5 | 0.573 (0.367–0.897) | 0.015 |  |  |
|  | Craniotomy/brain surgery | No | 1610 | 33 | 0.680 (0.600–0.771) | <0.001 | 0.066 | 0.158 |
|  |  | Yes | 611 | 14 | 0.536 (0.418–0.686) | <0.001 |  |  |
|  | IVC filter | No | 2179 | 45 | 0.649 (0.581–0.724) | <0.001 | NE | NE |
|  |  | Yes | 42 | 2 | NE | NE |  |  |

Hazard ratios are reported per 1-unit increase in PANI/1,000 and were adjusted using the Model 3 covariate set.

When the stratifying variable was itself included in Model 3, that variable was omitted from the corresponding subgroup-specific and interaction models.

P values for interaction were obtained from likelihood-ratio tests of multiplicative interaction terms.

Benjamini-Hochberg false discovery rate correction was applied separately within each outcome across evaluable interaction tests.

NE indicates not evaluable because of sparse events; the inferior vena cava filter interaction was excluded from multiplicity correction.

Because albumin is a direct multiplicative component of PANI (PANI = albumin × prealbumin), the PANI-by-albumin interaction represents structural mathematical coupling and should not be interpreted as biological effect modification.
