# Supplementary material for: Association of the prealbumin–albumin nutritional index with short-term mortality in hospitalized patients with traumatic brain injury
Source: Front Nutr. 2026 Sep 3;13:1910413. doi: 10.3389/fnut.2026.1910413 (PMC13581652; doi:10.3389/fnut.2026.1910413)
Supplement: Supplementary file 4 [file Table_4.DOCX]

**Table S4.** Bootstrap internal validation, calibration, and overall performance. Internal validation used 1,000 bootstrap resamples.

| **Outcome** | **Model** | **N/events** | **Apparent AUC** | **Optimism-corrected AUC** | **Apparent Brier score** | **Optimism-corrected Brier score** | **Corrected calibration intercept** | **Corrected calibration slope** | **Hosmer–Lemeshow chi-square (df)** | **Hosmer–Lemeshow P** | **Successful bootstrap samples** |
| --- | --- | --- | --- | --- | --- | --- | --- | --- | --- | --- | --- |
|  |  |  |  |  |  |  |  |  |  |  |  |
| In-hospital mortality | PANI-only | 2221/78 | 0.856 | 0.855 | 0.029 | 0.029 | 0.006 | 1.001 | 18.947 (8) | 0.015 | 1000 |
| In-hospital mortality | Baseline covariates | 2151/77 | 0.858 | 0.838 | 0.033 | 0.033 | -0.004 | 0.902 | 27.077 (8) | <0.001 | 1000 |
| In-hospital mortality | Baseline covariates + PANI | 2151/77 | 0.869 | 0.858 | 0.028 | 0.029 | -0.011 | 0.915 | 7.575 (8) | 0.476 | 1000 |
| 7-day in-hospital mortality | PANI-only | 2221/47 | 0.837 | 0.838 | 0.019 | 0.019 | 0.009 | 1.018 | 15.090 (8) | 0.057 | 1000 |
| 7-day in-hospital mortality | Baseline covariates | 2151/46 | 0.841 | 0.804 | 0.021 | 0.022 | 0.002 | 0.837 | 16.179 (8) | 0.040 | 1000 |
| 7-day in-hospital mortality | Baseline covariates + PANI | 2151/46 | 0.852 | 0.833 | 0.019 | 0.020 | -0.001 | 0.888 | 9.454 (8) | 0.305 | 1000 |

Discrimination, calibration, and decision-curve analyses were based on binary logistic regression models for in-hospital mortality and 7-day in-hospital mortality. These prediction analyses were supplementary to the primary time-to-event Cox regression analyses.

The baseline covariates were age, sex, diabetes, hypertension, other injury, alanine aminotransferase, creatinine, and international normalized ratio.

Lower Brier scores indicate better overall accuracy. Calibration intercepts closer to 0 and slopes closer to 1 indicate better calibration. The Hosmer–Lemeshow test was reported as an additional calibration measure; bootstrap-corrected calibration estimates were prioritized for interpretation.
