# Supplementary material for: Association of the prealbumin–albumin nutritional index with short-term mortality in hospitalized patients with traumatic brain injury
Source: Front Nutr. 2026 Sep 3;13:1910413. doi: 10.3389/fnut.2026.1910413 (PMC13581652; doi:10.3389/fnut.2026.1910413)
Supplement: Supplementary file 5 [file Table_5.DOCX]

**Table S5.** Complete-case sensitivity and within-tertile analyses. Panel A additionally incorporates BMI and systolic blood pressure; Panel B reports continuous PANI associations within each tertile.

**A. Model 4 plus BMI/SBP complete-case sensitivity model**

| **Outcome** | **Model** | **HR (95% CI)** | **P value** | **N/events** |
| --- | --- | --- | --- | --- |
| In-hospital mortality | Model 4 + BMI/SBP complete-case sensitivity model | 0.5620 (0.4661–0.6777) | <0.001 | 1102/27 |
| 7-day in-hospital mortality | Model 4 + BMI/SBP complete-case sensitivity model | 0.6772 (0.5275–0.8695) | 0.002 | 1102/12 |

**B. Within-tertile continuous analyses**

| **Outcome** | **Tertile** | **Model** | **HR (95% CI)** | **P value** | **N/events** |
| --- | --- | --- | --- | --- | --- |
| In-hospital mortality | Low tertile | Model 1 | 0.5468 (0.4772–0.6266) | <0.001 | 741/63 |
| In-hospital mortality | Low tertile | Model 2 | 0.5424 (0.4721–0.6233) | <0.001 | 741/63 |
| In-hospital mortality | Low tertile | Model 3 | 0.5292 (0.4585–0.6108) | <0.001 | 741/63 |
| In-hospital mortality | Middle tertile | Model 1 | 0.2840 (0.1036–0.7783) | 0.014 | 740/11 |
| In-hospital mortality | Middle tertile | Model 2 | 0.2879 (0.1028–0.8059) | 0.018 | 740/11 |
| In-hospital mortality | Middle tertile | Model 3 | 0.2933 (0.1033–0.8329) | 0.021 | 740/11 |
| In-hospital mortality | High tertile | Model 1 | 0.9598 (0.5956–1.5469) | 0.866 | 740/4 |
| In-hospital mortality | High tertile | Model 2 | 1.0539 (0.6531–1.7005) | 0.830 | 740/4 |
| In-hospital mortality | High tertile | Model 3 | 1.0631 (0.6711–1.6840) | 0.794 | 740/4 |
| 7-day in-hospital mortality | Low tertile | Model 1 | 0.5810 (0.4904–0.6885) | <0.001 | 741/38 |
| 7-day in-hospital mortality | Low tertile | Model 2 | 0.5858 (0.4937–0.6950) | <0.001 | 741/38 |
| 7-day in-hospital mortality | Low tertile | Model 3 | 0.5735 (0.4812–0.6834) | <0.001 | 741/38 |
| 7-day in-hospital mortality | Middle tertile | Model 1 | 0.4597 (0.1477–1.4306) | 0.180 | 740/6 |
| 7-day in-hospital mortality | Middle tertile | Model 2 | 0.4896 (0.1538–1.5587) | 0.227 | 740/6 |
| 7-day in-hospital mortality | Middle tertile | Model 3 | 0.5069 (0.1571–1.6351) | 0.255 | 740/6 |
| 7-day in-hospital mortality | High tertile | Model 1 | 1.0727 (0.6778–1.6975) | 0.765 | 740/3 |
| 7-day in-hospital mortality | High tertile | Model 2 | 1.2074 (0.7601–1.9180) | 0.425 | 740/3 |
| 7-day in-hospital mortality | High tertile | Model 3 | 1.1739 (0.7427–1.8557) | 0.492 | 740/3 |

Model 4 adjusted for age, sex, diabetes, hypertension, other injury, alanine aminotransferase, creatinine, and INR. The complete-case model additionally included BMI and SBP and is interpreted cautiously because of missing data and fewer events. Within-tertile estimates are continuous slopes within each tertile, not between-tertile comparisons; high-tertile estimates are especially imprecise because of sparse events.
