# Supplementary material for: Association of the prealbumin–albumin nutritional index with short-term mortality in hospitalized patients with traumatic brain injury
Source: Front Nutr. 2026 Sep 3;13:1910413. doi: 10.3389/fnut.2026.1910413 (PMC13581652; doi:10.3389/fnut.2026.1910413)
Supplement: Supplementary file 6 [file Table_6.DOCX]

**Table S6.** Missing data in the manuscript source cohort and final analytical cohort. The source cohort comprised 2,512 patients with an available first post-admission albumin measurement, consistent with Figure 1.

| **Variable** | **Missing n, source cohort (n=2,512)** | **Missing %, source cohort (n=2,512)** | **Missing n, final cohort (n=2,221)** | **Missing %, final cohort (n=2,221)** |
| --- | --- | --- | --- | --- |
|  |  |  |  |  |
| Age | 0 | 0.0% | 0 | 0.0% |
| Sex | 0 | 0.0% | 0 | 0.0% |
| Length of stay | 0 | 0.0% | 0 | 0.0% |
| In-hospital death | 0 | 0.0% | 0 | 0.0% |
| Systolic blood pressure | 720 | 28.7% | 614 | 27.6% |
| Diastolic blood pressure | 720 | 28.7% | 614 | 27.6% |
| Height | 119 | 4.7% | 109 | 4.9% |
| Weight | 677 | 27.0% | 626 | 28.2% |
| International normalized ratio | 90 | 3.6% | 70 | 3.2% |
| Alanine aminotransferase | 14 | 0.6% | 0 | 0.0% |
| Aspartate aminotransferase | 14 | 0.6% | 0 | 0.0% |
| Creatinine | 14 | 0.6% | 0 | 0.0% |
| Urea nitrogen | 14 | 0.6% | 0 | 0.0% |
| Albumin | 0 | 0.0% | 0 | 0.0% |
| Prealbumin | 290 | 11.5% | 0 | 0.0% |
| Lymphocyte count | 0 | 0.0% | 0 | 0.0% |
| Hemoglobin | 10 | 0.4% | 0 | 0.0% |
| Platelet count | 10 | 0.4% | 0 | 0.0% |
| Other injury | 0 | 0.0% | 0 | 0.0% |
| Diabetes | 0 | 0.0% | 0 | 0.0% |
| Hypertension | 0 | 0.0% | 0 | 0.0% |
| Tracheotomy | 0 | 0.0% | 0 | 0.0% |
| Mechanical ventilation | 0 | 0.0% | 0 | 0.0% |
| Craniotomy/brain surgery | 0 | 0.0% | 0 | 0.0% |
| Orthopedic surgery | 0 | 0.0% | 0 | 0.0% |
| Inferior vena cava filter | 0 | 0.0% | 0 | 0.0% |
| Body mass index | 677 | 27.0% | 626 | 28.2% |

Body mass index was derived from available height and weight. Missing counts in the source cohort may overlap; the flowchart exclusions were applied hierarchically, first for missing prealbumin and then for other required index components.
