# Supplementary material for: Association of the prealbumin–albumin nutritional index with short-term mortality in hospitalized patients with traumatic brain injury
Source: Front Nutr. 2026 Sep 3;13:1910413. doi: 10.3389/fnut.2026.1910413 (PMC13581652; doi:10.3389/fnut.2026.1910413)
Supplement: Supplementary file 7 [file Table_7.DOCX]

**Table S7.** Restricted cubic spline robustness analyses of the association between PANI/1,000 and mortality.

| **Outcome** | **Analysis** | **Knot specification** | **N/events** | **Spline df** | **Nonlinear df** | **Overall LRT χ²** | **P overall** | **Nonlinearity LRT χ²** | **P nonlinear** |
| --- | --- | --- | --- | --- | --- | --- | --- | --- | --- |
| In-hospital mortality | 3-knot RCS, full cohort | 10th/50th/90th percentiles: 4.7601, 8.9761, 13.5048 | 2221/78 | 2 | 1 | 147.451 | <0.001 | 7.003 | 0.008 |
|  | Exploratory 4-knot RCS, full cohort | 5th/35th/65th/95th percentiles: 3.5200, 7.7621, 10.2018, 15.2220 | 2221/78 | 3 | 2 | 148.199 | <0.001 | 7.752 | 0.021 |
|  | 3-knot RCS, influential event excluded | Same 3 knots as full cohort: 4.7601, 8.9761, 13.5048 | 2220/77 | 2 | 1 | 153.325 | <0.001 | 0.562 | 0.453 |
| 7-day in-hospital mortality | 3-knot RCS, full cohort | 10th/50th/90th percentiles: 4.7601, 8.9761, 13.5048 | 2221/47 | 2 | 1 | 80.321 | <0.001 | 5.628 | 0.018 |
|  | Exploratory 4-knot RCS, full cohort | 5th/35th/65th/95th percentiles: 3.5200, 7.7621, 10.2018, 15.2220 | 2221/47 | 3 | 2 | 81.538 | <0.001 | 6.844 | 0.033 |
|  | 3-knot RCS, influential event excluded | Same 3 knots as full cohort: 4.7601, 8.9761, 13.5048 | 2220/46 | 2 | 1 | 86.227 | <0.001 | 0.038 | 0.846 |

All RCS models were adjusted using the Model 3 covariate set: age, sex, diabetes, hypertension, tracheotomy, mechanical ventilation, craniotomy/brain surgery, and inferior vena cava filter placement.

Three knots were placed at the 10th, 50th, and 90th percentiles; four knots were placed at the 5th, 35th, 65th, and 95th percentiles.

The influence analysis excluded the single in-hospital death with the highest PANI/1,000 and retained the same full-cohort 3-knot locations.

The reference PANI/1,000 was the full-cohort median. The exploratory four-knot findings are retained for transparency but are not interpreted as evidence of a biological or clinical threshold.
