# Supplementary material for: Association of the prealbumin–albumin nutritional index with short-term mortality in hospitalized patients with traumatic brain injury
Source: Front Nutr. 2026 Sep 3;13:1910413. doi: 10.3389/fnut.2026.1910413 (PMC13581652; doi:10.3389/fnut.2026.1910413)
Supplement: Supplementary file 8 [file Table_8.DOCX]

**Table S8.** Comparison of patients with available versus missing prealbumin measurements in the source cohort.

| **Variable** | **Prealbumin available (n=2,222)** | **Available n** | **Prealbumin missing (n=290)** | **Available n (missing group)** | **SMD** | **P value** |
| --- | --- | --- | --- | --- | --- | --- |
| Age, years | 50.55 ± 17.34 | 2222 | 46.92 ± 22.82 | 290 | 0.179 | 0.009 |
| Length of stay, days | 17.05 ± 17.72 | 2222 | 8.02 ± 7.64 | 290 | 0.662 | <0.001 |
| Albumin, g/L | 38.49 ± 6.52 | 2222 | 37.68 ± 9.50 | 290 | 0.100 | 0.159 |
| Hemoglobin | 125.84 ± 25.08 | 2221 | 118.91 ± 34.55 | 281 | 0.230 | 0.001 |
| Platelet count | 185.87 ± 72.71 | 2221 | 188.31 ± 82.28 | 281 | 0.031 | 0.635 |
| Alanine aminotransferase | 29.48 ± 37.41 | 2222 | 32.56 ± 36.49 | 276 | 0.083 | 0.188 |
| Aspartate aminotransferase | 37.32 ± 43.64 | 2222 | 51.17 ± 64.41 | 276 | 0.252 | <0.001 |
| Creatinine | 64.12 ± 40.20 | 2222 | 63.75 ± 30.69 | 276 | 0.010 | 0.856 |
| Urea nitrogen | 5.25 ± 2.08 | 2222 | 5.39 ± 1.85 | 276 | 0.071 | 0.243 |
| International normalized ratio | 1.03 ± 0.18 | 2152 | 1.09 ± 0.28 | 270 | 0.258 | <0.001 |
| Male sex | 1590 (71.6%) | 2222 | 198 (68.3%) | 290 | 0.072 | 0.246 |
| Other injury | 260 (11.7%) | 2222 | 33 (11.4%) | 290 | 0.010 | 0.872 |
| Diabetes | 110 (5.0%) | 2222 | 19 (6.6%) | 290 | 0.069 | 0.245 |
| Hypertension | 260 (11.7%) | 2222 | 40 (13.8%) | 290 | 0.063 | 0.302 |
| Tracheotomy | 286 (12.9%) | 2222 | 36 (12.4%) | 290 | 0.014 | 0.827 |
| Mechanical ventilation | 162 (7.3%) | 2222 | 23 (7.9%) | 290 | 0.024 | 0.695 |
| Craniotomy/brain surgery | 611 (27.5%) | 2222 | 96 (33.1%) | 290 | 0.122 | 0.046 |
| Inferior vena cava filter | 42 (1.9%) | 2222 | 9 (3.1%) | 290 | 0.078 | 0.168 |
| In-hospital mortality | 78 (3.5%) | 2222 | 40 (13.8%) | 290 | 0.372 | <0.001 |
| 7-day in-hospital mortality | 47 (2.1%) | 2222 | 32 (11.0%) | 290 | 0.366 | <0.001 |

This comparison is restricted to the manuscript source cohort of 2,512 patients with available albumin.

Continuous variables are shown as mean ± SD; categorical variables are shown as n (%). SMD denotes absolute standardized mean difference.

The comparison is descriptive and is intended to assess possible selective prealbumin measurement and selection bias; it does not establish the reason for missing prealbumin testing or the direction of bias in the PANI-mortality association.
